# The shape and size of hydroxyapatite particles dictate inflammatory responses following implantation

**DOI:** 10.1038/s41598-017-03086-0

**Published:** 2017-06-07

**Authors:** Filipa Lebre, Rukmani Sridharan, Michael J. Sawkins, Daniel J. Kelly, Fergal J. O’Brien, Ed C. Lavelle

**Affiliations:** 1 Adjuvant Research Group, School of Biochemistry and Immunology, Trinity Biomedical Sciences Institute, Trinity College Dublin, Dublin 2, D02 R590 Ireland; 2 Trinity Centre for Bioengineering, Trinity Biomedical Sciences Institute, Trinity College Dublin, Dublin 2, D02 R590 Ireland; 3 Tissue Engineering Research Group, Department of Anatomy, Royal College of Surgeons in Ireland, Dublin 2, Ireland; 4 Centre for Research on Adaptive Nanostructures and Nanodevices (CRANN), Trinity College Dublin, Dublin 2, D02 PN40 Ireland; 5 Advanced Materials Bio-Engineering Research Centre (AMBER), Trinity College Dublin, Dublin 2, D02 PN40 Ireland

## Abstract

The extent of regeneration following biomaterial implantation is dependent on the microenvironment surrounding the implant. Since implant composition can have a profound effect on inflammation, it is essential to understand this process as a non-resolving inflammatory response can lead to fibrous encapsulation and insufficient integration. Incorporation of particulates into implants confers structural and functional benefits, thus optimizing particulate characteristics to enhance immune mediated efficacy is important. We investigated the relationship between the nature of hydroxyapatite (HA) particles and the innate immune response, focusing on how particle size (0.1 µm, 5 µm, 20 µm, 100 µm) and morphology (needle-shaped/spherical; smooth/rough surface) modulates inflammatory responses. We observed a shape and size-dependent activation of the NLRP3 inflammasome and IL-1β secretion; while needle-shaped and smaller HA particles significantly enhanced cytokine secretion, larger particles did not. Moreover, HA particle characteristics profoundly influenced patterns of innate immune cell recruitment and cytokine production following injection. While small, needle-shaped particles induced a strong inflammatory response, this was not observed with smooth, spherical particles of comparable size or with larger particles. These findings indicate that hydroxyapatite particle characteristics dictate immune cell recruitment and the ensuing inflammatory response, providing an opportunity to tailor HA particle characteristics to regulate immune responses induced after biomaterial implantation.

## Introduction

Due to an increasingly ageing population and an exponential growth in lifestyle-related diseases, the use of biomaterials to replace or regenerate damaged tissue is steadily rising^1^. The first generation of biomaterial-based implants were developed with the consensus that the best biological performance would be achieved with materials that did not engage immune responses, such as metallic implants and non-degradable synthetic polymers^2, 3^. However, in recent decades, huge strides have been made in understanding the interactions between implanted materials and host cells^4, 5^. As a result, tissue repair approaches have grown in sophistication, with the advent of several natural and synthetic materials that interact with host cells to enhance tissue repair and regeneration. In this context, while the composition of materials has been explored extensively, it is only recently that biophysical and biochemical cues have been implicated in driving tissue regeneration. For instance, it is now established in the tissue engineering field that biomaterial properties such as stiffness and topography can direct the differentiation of mesenchymal stem cells (MSC) into mature cells of different tissue specific lineages^6, 7^. By designing biomaterials with tailored specific properties, it may be possible to direct the differentiation of host stem cells into a pre-determined lineage to promote successful tissue integration.

Cells of the innate immune system (e.g. macrophages, dendritic cells, neutrophils) are now perceived to be the first responders to implantation of a biomaterial^8^, with the phenotype of these cells modulated by the structure and composition of the implant. Dendritic cells (DC), for example, recognize danger-associated molecular patterns (DAMPs) produced by other host cells that are damaged, stressed or necrotic in response to implantation^9^. Moreover, it has been shown that the type of biomaterial implanted can dictate the maturation of dendritic cells, necessary to activate the adaptive immune system, with materials like chitosan inducing maturation, an effect not observed with alginate and agarose^10, 11^. Additionally, a strong and sustained macrophage response is also observed around implanted materials^12^, and macrophages have been implicated in the foreign body response to biomaterials^13^, highlighting the role of macrophages in the outcome of the regenerative process following implantation. Even though inflammation is generally associated with adverse outcomes (e.g. foreign body reaction, implant rejection), it is established that pro-inflammatory signals secreted by immune cells are important in early stages of the normal tissue repair process, provided that they resolve over time^14^. *In situ* tissue engineering approaches can influence the microenvironment surrounding tissue engineered constructs to improve the outcome of implantation. In this context, the presence of specific immune cell populations (including neutrophils, mast cells, monocytes and macrophages) following implantation may be predictive of implant efficacy^8^. However, the relative contribution of each population and the balance needed between them to achieve scaffold integration and vascularization is unclear. Further research is therefore essential to carefully delineate the relationship between material characteristics, the ensuing immune response and subsequent implant integration.

Nano- and microparticles are widely used for coating of metallic implants^15^, controlled delivery of therapeutic molecules such as genes and proteins to tissues of interest^16^, vaccine delivery^17, 18^ and for *in vivo* imaging^19^. Despite the progress made in fabrication of particles of different sizes, morphologies and chemical properties, the mechanisms underlying how particle properties modulate immune responses have not been fully resolved. Several studies have focused on the effect of particle size on immune cells *in vitro* ^20, 21^, with limited follow up in an *in vivo* setting. Furthermore, fabrication of micro- and nanoparticles in the laboratory often results in endotoxin contamination. Hence, studies that report immune responses to biomaterials should first test the materials for endotoxins to ensure that the responses seen from immune cells are not altered due to their presence, and should further confirm this by using cells that do not respond to endotoxins^22, 23^. Given the broad range of applications of nano- and microparticles, there is hence a critical need for a more detailed understanding of the immune response to these particles.

This study focuses on hydroxyapatite, which is widely used in the bone tissue engineering field because of its osteogenic capabilities^24, 25^. Here we describe the immune responses induced by hydroxyapatite particles of defined sizes and morphologies (**S**mooth spherical particles with mean size 0.1 μm − S_0.1_, Needle-shaped particles with mean size 5 μm − N_5_, **S**mooth or Rough particles with mean size 20 μm − S_20_ and R_20_ respectively, and **S**mooth particles with mean size 100 μm − S_100_). We addressed cytotoxicity and particle-induced cytokine production by dendritic cells and macrophages and specifically examined the role of the NLRP3 inflammasome in this context. Since the polydispersity of particulate preparations can make it difficult to attribute differential responses to specific particle characteristics, we refined particle sizes by sieving to evaluate the size-dependent production of IL-1β by murine bone marrow derived dendritic cells (BMDC). Finally, we determined the importance of size and shape in the magnitude and type of inflammatory responses triggered following injection of particles in mice. Small-needle shaped hydroxyapatite particles induced the greatest inflammatory response, while limited responses were triggered by similar sized spherical particles or larger particles, particularly in macrophages.

## Results

### The physical properties of hydroxyapatite particles dictate cytokine production by murine dendritic cells

**Figure 1:**
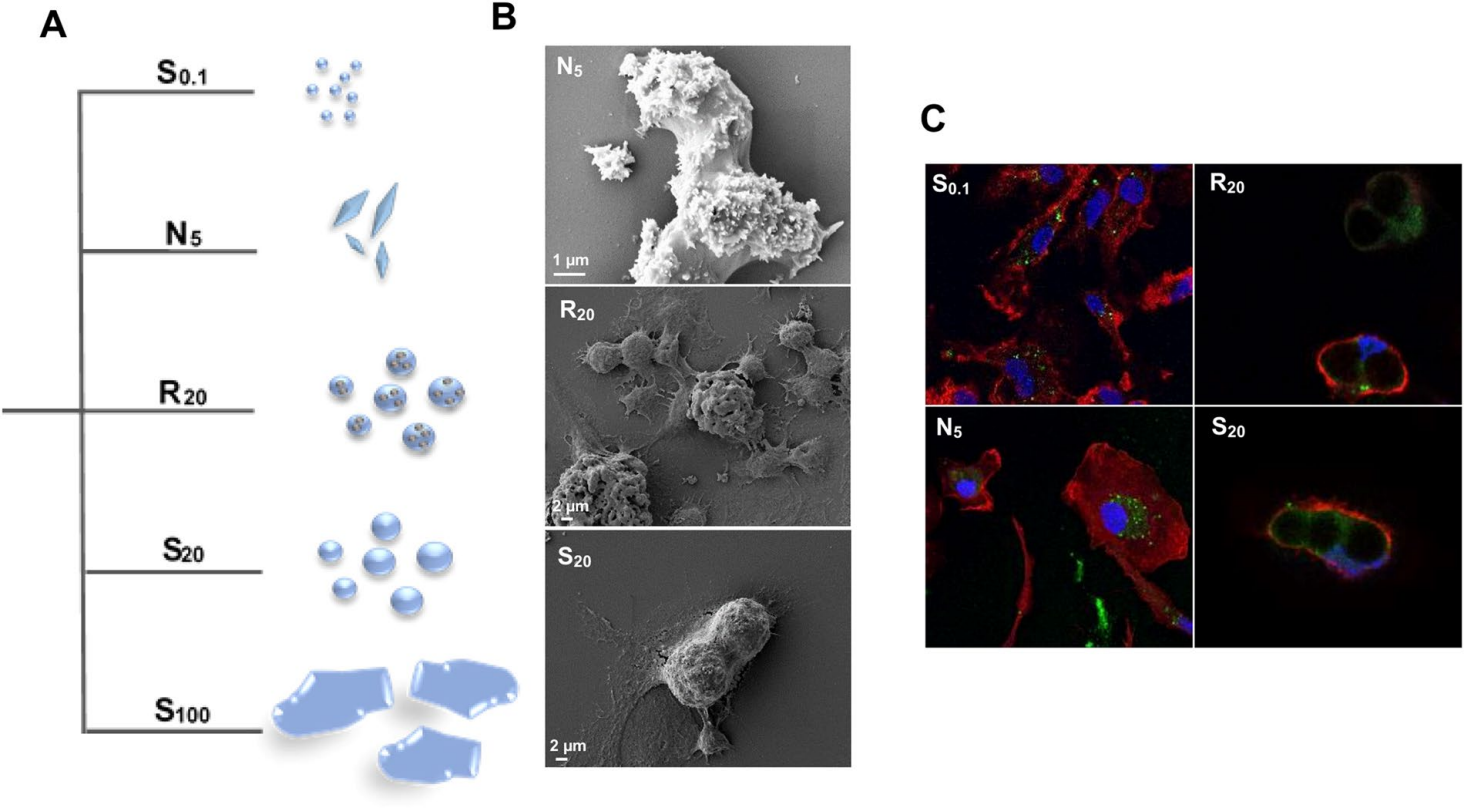
(**A**) Schematic of hydroxyapatite particles. (**B**) HA internalization by BMDCs. SEM images of BMDCs incubated with N_5_, R_20_ and S_20_ particles for 18 h. (**C**) Confocal fluorescence microscopy images of BMDCs incubated with FITC-labelled (green) S_0.1_, N_5_, R_20_ and S_20_ for 24 h to visualize the individual internalized particles. Membrane staining with phalloidin–TRITC (red) and nuclear staining with DAPI (blue).

**Figure 2:**
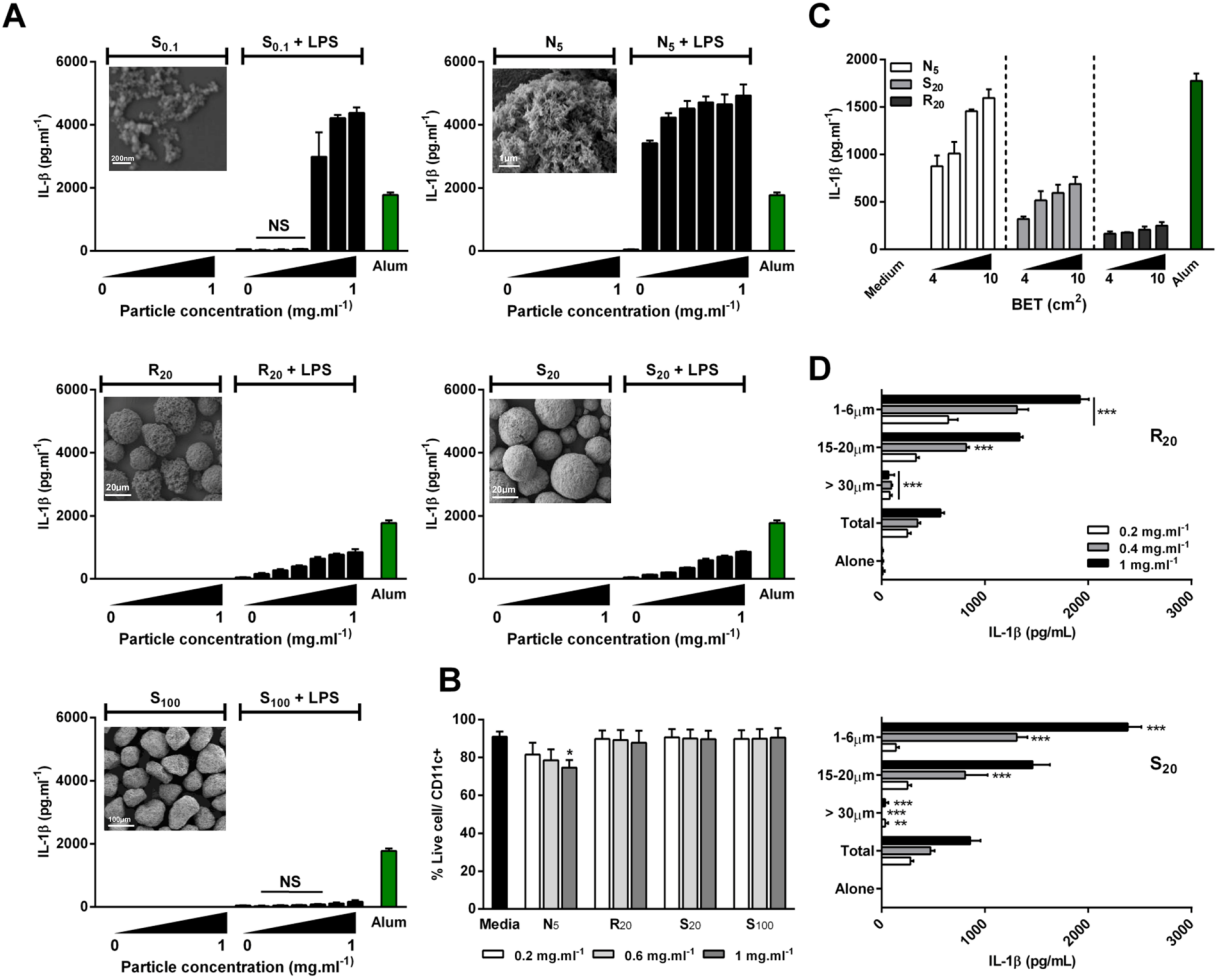
Hydroxyapatite particle size and shape dictates cytokine production by murine BMDCs. BMDCs (0.625 × 10^6^ cells · ml^−1^) from C57BL/6 mice were stimulated with concentrations ranging from 0.1 mg · ml^−1^ to 1 mg · ml^−1^ alone or after priming with LPS (1 ng · ml^−1^) for 3 h. Supernatants were collected 24 hours later and tested for IL-1β (**A**,**C**,**D**) by ELISA. Unprimed cells were collected and stained with LIVE/DEAD^®^ fixable Aqua dead cell stain and CD11c^+^ and cell death was assessed by flow cytometry (**B**). Representative SEM pictures of HA particles (**A**). Results are mean cytokine concentrations (±SD) for triplicate samples ((**A**) vs LPS alone; (**D**) vs Total, NS p > 0.05, *p < 0.05 and ***p < 0.001). Data are representative of three independent experiments. (**B**) Results are pooled from three independent experiments. Error bars show means ± SEM for triplicate samples (vs media *p < 0.05); NS: non-significant.

Nano-sized HA particles (S_0.1_) and micro-sized HA particles (N_5_, S_20_, R_20_ and S_100_) (Fig. 1) were characterized for size (dynamic light scattering), ζ-potential (electrophoretic light scattering), surface area (Brunauer, Emmett and Teller (BET) analysis) (Table 1) and morphology (scanning electron microscopy (SEM)) (Fig. 2A (insets)). S_0.1_ particles are approximately 157 nm in size and have a spherical and smooth morphology; N_5_ particles are needle-shaped with a mean size of 5 μm; S_20_ and R_20_ have a mean size around 20 μm and differ in surface topography with S_20_ exhibiting a smooth surface and R_20_ a rough porous surface; the S_100_ particles have a mean size around 100 μm and have a smooth surface (Table 1).

**Table 1:** Hydroxyapatite particle characterization.

	d (0.1) (µm)	d (0.5) (µm)	d (0.9) (µm)	Mean size (nm)	ζ (mV)	Surface Area (m²/g)	Synthesis
S_0.1_	—	—	—	157.0 ± 0.80	−22.2 ± 0.90	—	Precipitation
N_5_	2.11	5.21	25.92	—	−20.9 ± 0.48	0.88	Precipitation
R_20_	12.79	22.80	39.35	—	−23.4 ± 2.23	2.60	Oven sintered
S_20_	13.70	21.63	33.81	—	−23.4 ± 2.73	1.12	Sintered + spray dried
S_100_	73.06	100.40	138.15	—	−23.8 ± 4.56	18.75	Sintered

Size and zeta potential of HA particles were measured by dynamic light scattering and electrophoretic light scattering, respectively. d (0.1), d (0.5) and d (0.9) indicate that 10 %, 50 % and 90 % of the particles lie below the values described.

We sought to evaluate the immunostimulatory potential of HA particles in BMDCs and bone-marrow derived macrophages (BMDMs), established models for assessing *in vitro* responses to biomaterials. Endotoxin contamination is a fundamental barrier that may contribute to documented immunostimulatory effects of some biomaterials. To exclude the possibility that the results seen here were a consequence of possible endotoxin contamination, HA particles were incubated with BMDCs and assessed for the secretion of the inflammatory cytokine IL-6, which is a highly sensitive readout for the presence of endotoxins^26^. HA particles alone did not promote the secretion of IL-6; moreover, they did not alter lipopolysaccharide (LPS)-induced secretion of this cytokine in the range of concentrations tested (0.1 mg · ml^−1^ to 1 mg · ml^−1^) (Fig. S1A). Likewise, using the *Limulus amebocyte* lysate (LAL) assay, HA particles alone did not contain detectable endotoxin (Fig. S1B).

In order to compare the various formulations, we performed uptake studies. SEM and confocal microscopy revealed that all HA particles, aside from S_100_, were effectively internalized by DC (Fig. 1B,C).

To determine the relationship between particle physicochemical characteristics and the enhancement of IL-1β, dendritic cells were incubated with a range of concentrations (0.1 mg · ml^−1^ to 1 mg · ml^−1^) of HA particles alone or in combination with the Toll-like receptor (TLR)-4 agonist, LPS (1 ng · ml^−1^), and supernatants were analyzed for IL-1β secretion after 24 hours. LPS was used to prime the cells 3 hours before treatment with particles in order to trigger the NF-κB-dependent transcription of pro-IL-1β. In all groups, incubating HA particles with BMDC failed to elicit appreciable IL-1β levels, similar to cells in control media. However, significant differences in the secretion of IL-1β were observed between groups in cells primed with LPS (Fig. 2A). Overall, physical properties such as size and morphology decisively influenced the magnitude of IL-1β secretion, whereas surface topography (S_20_ vs. R_20_) did not significantly impact IL-1β secretion. Of the tested formulations, the needle-shaped N_5_ particles were the most potent in inducing IL-1β secretion, followed by the small S_0.1_, rough R_20_ and smooth S_20_ particles, with the largest S_100_ particles failing to enhance IL-1β secretion over treatment with LPS alone. Interestingly, low concentrations of N_5_ were sufficient to induce robust secretion of IL-1β in DCs with a dose dependent response observed with increase in particle concentration. Similarly, R_20_ and S_20_ particles both trigger a dose dependent IL-1β secretion at all tested concentrations, but at moderate levels when compared to N_5_ particles. While S_0.1_ particles induced significantly higher levels of IL-1β secretion compared to R_20_ and S_20_ at concentrations higher than 0.4 mg · ml^−1^, this was not observed for lower concentrations. Alum (500 mg · ml^−1^), a well-established NLRP3 inflammasome activator, was used as a positive control.

Innate immune cells respond to DAMPs which are released in response to stress, tissue damage and necrotic cell death^27^. Therefore, and in order to exclude toxicity as a key factor mediating cytokine release profile, the cytotoxic effect of HA particles was assessed. BMDCs were incubated with increasing concentrations of HA particles; cells were harvested after 24 hours, stained to assess viability and analyzed by flow cytometry. With the exception of the N_5_ formulation, the particles induced negligible cell death, with only the highest concentration of N_5_ inducing significantly higher cell death when compared to cells in culture medium (Fig. 2B).

Since differences in surface area can confound interpretation of the effects of particle size on immune cell activation, particle dose was normalized to Brunauer, Emmett and Teller (BET) surface area to ensure that the distinct inflammatory profiles generated by HA particles were not due to differences in surface area. When analysed on the basis of particle surface area, N_5_ particles resulted in the highest IL-1β secretion, followed by S_20_, with R_20_ particles promoting the least IL-1β secretion (Fig. 2C).

Finally, to further elucidate the effect of particle size in HA-induced IL-1β secretion, the polydispersity of the R_20_ and S_20_ particles was reduced by separating them into distinct size distributions using a set of sieves with variable mesh sizes (Fig. S2). Formulations were successfully separated into three distinct fractions: >30 µm, 15–20 μm and 1–6 μm. BMDCs were incubated with increasing concentrations (0.2 mg · ml^−1^ to 1 mg · ml^−1^) of each fraction for both formulations and compared with the non-sieved formulation (total). Figure 2D illustrates the size-dependency in IL-1β secretion in response to sieved S_20_ and R_20_ particles. BMDCs secreted significantly more IL-1β in response to the smallest particles (1–6 μm), which was evident in both the R_20_ and S_20_ groups. Moreover, cytokine secretion levels were comparable between both formulations for the range of sizes tested, demonstrating that surface topography does not significantly impact on IL-1β release from BMDCs. Particles larger than 30 μm failed to induce appreciable secretion of IL-1β compared to their smaller counterparts for both formulations. Interestingly, fractions containing smaller particles (15–20 µm and 1–6 µm) induced higher IL-1β secretion compared to the non-sieved formulation. In order to confirm the importance of shape, N_5_ particles were also sieved; however, due to their morphology, the sieving process was not as efficient as for spherical particles. Nevertheless, when directly compared, IL-1β secretion was significantly higher for cells stimulated with the 1–6 μm fraction of the needle-shaped particles compared to smooth or rough particles (Fig. S3), strongly suggesting that shape is a key determinant of HA particle-induced inflammasome activation.

### Needle-shaped but not spherical HA particles potently enhance IL-1β secretion by macrophages

**Figure 3:**
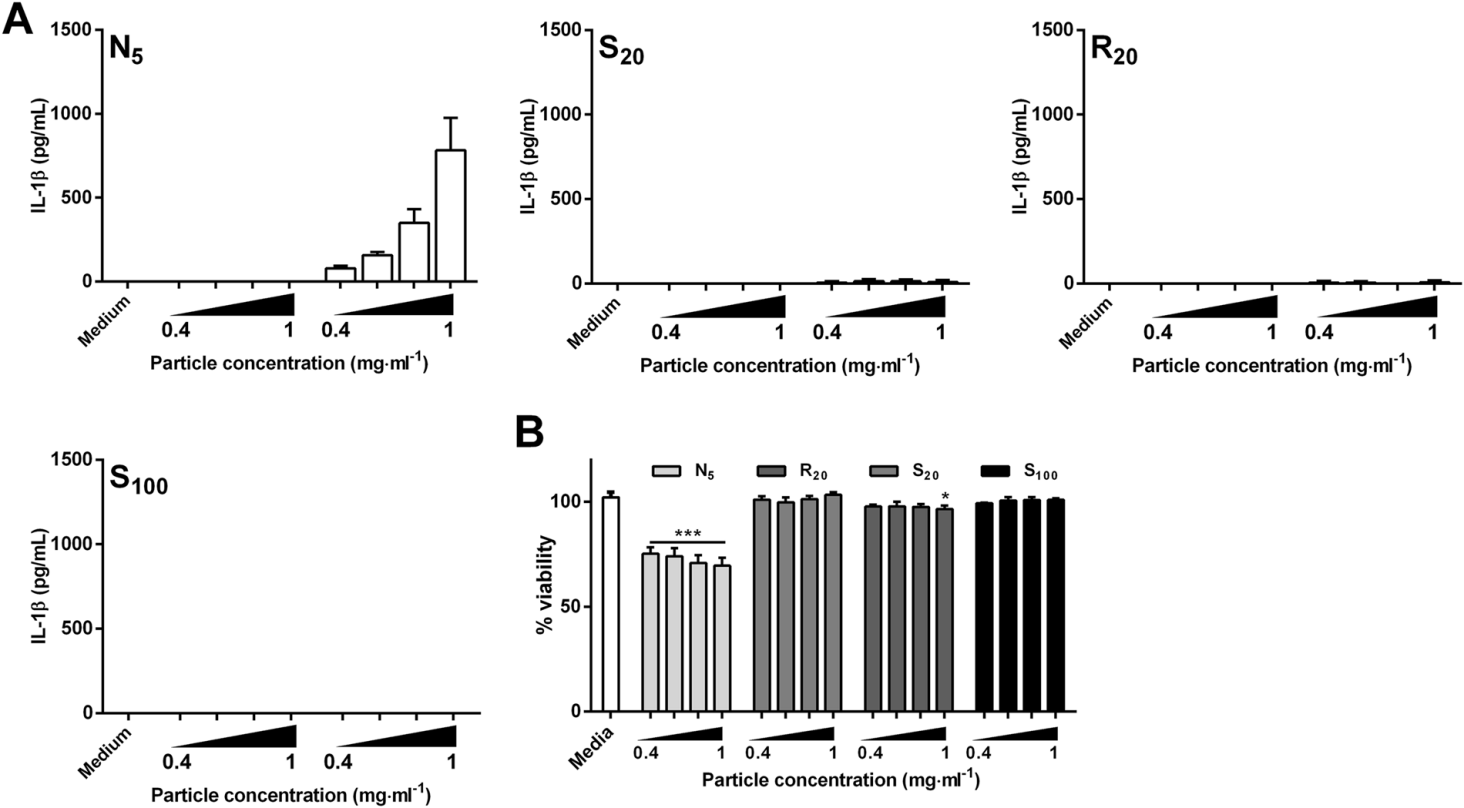
Hydroxyapatite particle shape dictates cytokine production by murine BMDMs. BMDMs (1.0 × 10^6^ cells · ml^−1^) from C57BL/6 mice were stimulated with particles at concentrations ranging from 0.4 mg · ml^−1^ to 1 mg · ml^−1^ alone or after priming with LPS (10  ng · ml^−1^) for 3 h. Supernatants were collected 24 hours later and tested for (**A**) IL-1β secretion by ELISA or (**B**) LDH release to assess cell viability. (**A**) Results are mean cytokine concentrations (±SD) for triplicate samples (vs LPS alone NS p > 0.05, *p < 0.05 and ***p < 0.001). Data are representative of three independent experiments. (**B**) Error bars show means ± SD for triplicate samples (vs media *p < 0.05 and ***p < 0.001). Results are representative of two independent experiments.

Following our observations in BMDCs, we sought to evaluate IL-1β secretion by BMDMs, since macrophages are important players in the outcome of biomaterial implantation^28^. BMDMs were incubated with a range of concentrations (0.4 mg · ml^−1^ to 1 mg · ml^−1^) of HA particles alone or after 3 h priming with LPS (10 ng · ml^−1^). Similar to DCs, particles alone failed to induce cytokine production (Fig. 3A). Strikingly, HA particle-induced enhancement of IL-1β production was only observed with the N_5_ particles. Unlike the needle-shaped particles, spherical particles (S_20_, R_20_ and S_100_), did not induce detectable IL-1β secretion, implying that shape plays a decisive role in HA particle-driven IL-1β secretion by macrophages. This may be related to the significant decrease in viability of BMDMs incubated with N_5_ particles for all concentrations tested, compared to the untreated cells, suggesting that the needle - shaped particles were cytotoxic (Fig. 3B). To further affirm the role of shape in the increased IL-1β secretion by BMDMs, we incubated the 1–6 μm fraction of the spherical S_20_ and R_20_ particles with the cells, and detected significantly lower levels of IL-1β secretion compared to needle shaped particles (Fig. S4).

### HA particles promote secretion of IL-1β in a NLRP3-dependent manner

**Figure 4:**
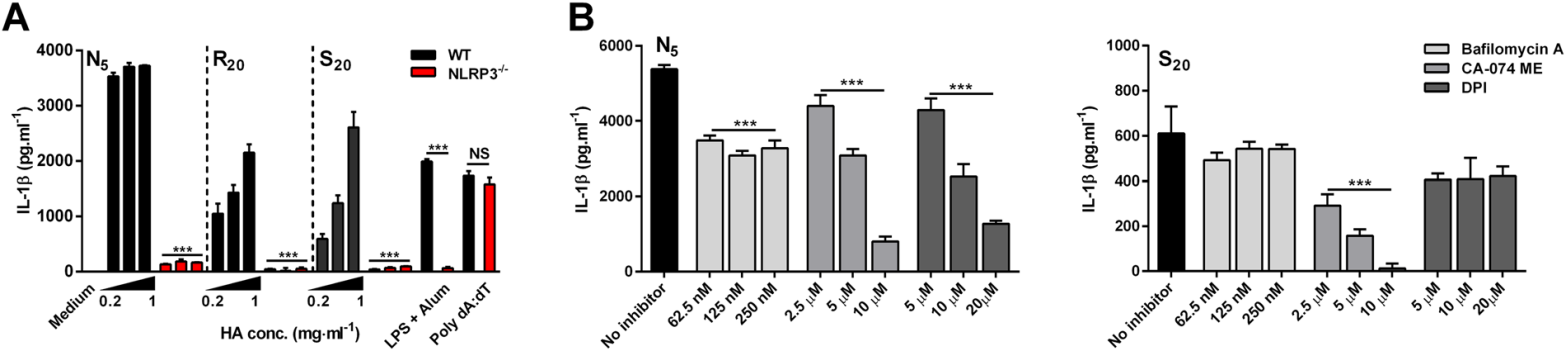
Hydroxyapatite particle-induced IL-1β production is NLRP3 dependent. (**A**) BMDCs (0.625 × 10^6^ cells · ml^−1^) from C57BL/6 or NLRP3^−/−^ mice were stimulated with HA particles at concentrations ranging from 0.2 mg · ml^−1^ to 1 mg · ml^−1^ after priming with LPS (1 ng · ml^−1^) for 3 h. Supernatants were collected after 24 hours and IL-1β levels were measured by ELISA. Data are shown as mean ± SD for triplicate samples (vs WT ***p < 0.001; NS: non-significant). Results are representative of three independent experiments. (**B**) BMDCs (0.625 × 10^6^ cells · ml^−1^) from female C57BL/6 mice were primed with LPS (1 ng · ml^−1^) for 3 h before stimulating with 0.8 mg · ml^−1^ of HA particles; cells were incubated with the lysosome acidification inhibitor bafilomycin A (62.5–250 nM), the cathepsin B inhibitor CA-074-Me (2.5–10 µM) and with the ROS inhibitor DPI (5–20 µM) 1 h before particulate addition. Supernatants were collected after 24 hours and IL-1β levels were measured by ELISA. Data are shown as mean ± SD for triplicate samples. Results are representative of three independent experiments (vs no inhibitor ***p < 0.001).

The ability of phosphocalcic crystals to activate the NLRP3 inflammasome has been previously reported^29^, but the relative contribution of size, shape and porosity has not been addressed in this context. Consistent with results presented in Fig. 2A, incubation of LPS-primed BMDCs with HA particles resulted in enhanced secretion of IL-1β (Fig. 4A); however, in contrast to BMDCs derived from wild type (WT) mice, HA particles did not enhance IL-1β secretion by NLRP3^−/−^ BMDCs. In line with previous observations, alum (500 mg · ml^−1^) induced significantly enhanced IL-1β secretion by WT, but not NLRP3^−/−^ BMDCs^30^. As a control, cells were transfected with the double stranded-DNA analog poly(deoxyadenylic-deoxythymidylic) (poly(dA:dT)), an AIM2 inflammasome agonist, which stimulated robust secretion of IL-1β in both cell types, confirming that the secretion of IL-1β by BMDC in response to particles was indeed NLRP3 dependent (Fig. 4A).

To understand the upstream events involved in hydroxyapatite particle–induced activation of the NLRP3 inflammasome, BMDCs were pre-treated with one of three inhibitors: Bafilomycin A, a V-ATPase inhibitor that blocks lysosome acidification; CA-074 ME, a Cathepsin B inhibitor and the NADPH oxidase inhibitor diphenyleneiodonium chloride (DPI), before the addition of either N_5_ or S_20_ formulations (0.8 mg · ml^−1^). While a significant dose-independent decrease in IL-1β production with N_5_ particles was observed when inhibiting lysosome acidification, this was not true for S_20_ (Fig. 4B). Inhibiting cathepsin B led to a dose-dependent decrease in IL-1β secretion for both N_5_ and S_20_ particles. Interestingly, pretreatment with DPI inhibited IL-1β secretion in a dose-dependent fashion only for N_5_ particles, but had no effect in response to S_20_ particles. These inhibitors did not enhance or reduce LPS/particle induced IL-6 secretion in these assays (data not shown).

### Needle-shaped HA particles induced significantly higher inflammatory responses than their spherical counter-parts after intraperitoneal injection

**Figure 5:**
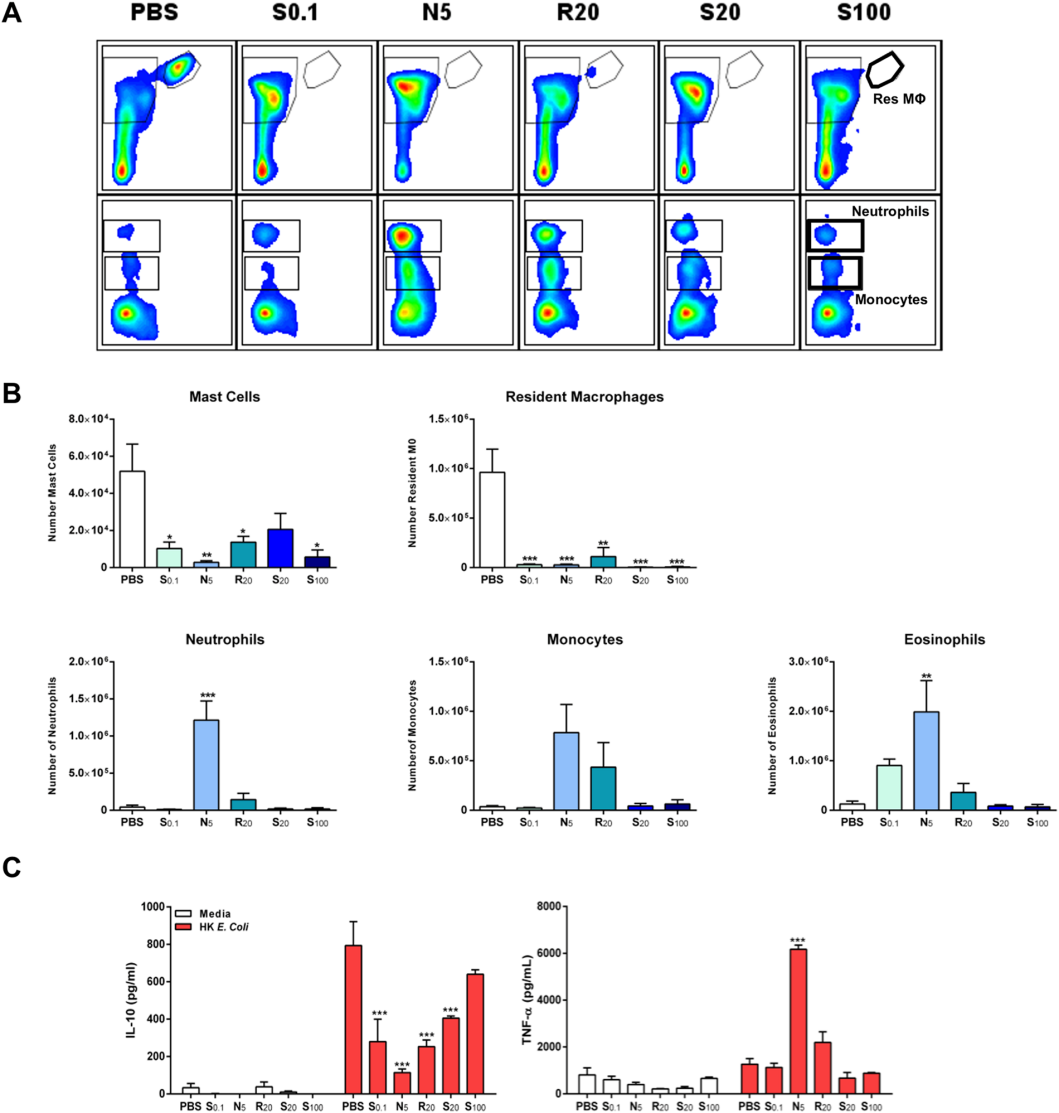
Size and shape dictates HA particle induced modulation of cytokine production following injection. Female C57BL/6 mice (n = 3) aged 6–8 weeks were injected intraperitoneally with PBS vehicle, S_0.1_ (1 mg/mouse), N_5_ (1 mg/mouse), R_20_ (1 mg/mouse), S_20_ (1 mg/mouse) or S_100_ (1 mg/mouse) HA particles, and 24 h later, peritoneal lavage cells were collected. (**A**,**B**) PECs were stained to analyse cell recruitment by flow cytometry. Cells were characterized using a combination of markers for peritoneal resident macrophages (CD11b^hi^ F4/80^hi^), mast cells (SiglecF^−^ cKit^+^) monocytes (CD11b^+^ SiglecF^−^ F4/80^−^ Gr1^int^ SSC^low^), neutrophils (CD11b^+^ SiglecF^−^ F4/80^−^ Gr1^hi^) and eosinophils (CD11b^+^ SiglecF^+^ Gr1^−^ SSC^hi^). Results are representative of two independent experiments. (**A**) Representative plots (**B**) Data are expressed as number of single/live cells. (*p < 0.05, **p < 0.01, ***p < 0.001). (**C**) PECs were restimulated with complete RPMI 1640 medium or heat-killed *Escherichia coli* (10 bacteria/cell) for 24 h. Supernatants were assessed for IL-10 and TNF-α secretion by ELISA. Results are mean cytokine concentrations (±SEM) for triplicate samples (vs PBS ***p < 0.001).

In order to translate the *in vitro* data to a more physiologically relevant *in vivo* system, we determined the innate immune response to HA particles after intraperitoneal injection. Mice were injected with PBS alone or in combination with 1 mg of each HA particle formulation. After 24 hours, the mice were sacrificed and the peritoneal exudate cells were collected and analyzed using flow cytometry. As shown in Fig. 5, PBS injected mice exhibited high numbers of resident peritoneal macrophages (CD11b^hi^ F4/80^hi^), moderate numbers of mast cells (SiglecF^−^ cKit^+^) and low numbers of monocytes (CD11b^+^ SiglecF^−^ F4/80^−^ Gr1^int^), neutrophils (CD11b^+^ SiglecF^−^ F4/80^−^ Gr1^hi^) and eosinophils (CD11b^+^ Gr1^low^ SiglecF^+^ cKit^−^). Twenty-four hours post-injection, the total number of resident macrophages was strikingly reduced compared to PBS treated mice in mice injected with all formulations. In contrast to the depletion of resident macrophages, we observed a significantly enhanced recruitment of neutrophils and eosinophils in the peritoneum of mice administered with N_5_ particles. Interestingly, even though R_20_ and S_0.1_ particles elicited some cell recruitment, this effect was not significant, suggesting that *in vivo*, hydroxyapatite particle shape plays a pivotal role in dictating the extent of immune cell infiltration.

We also determined whether HA particle injection impacted on the type or extent of cytokine produced. Cells isolated from peritoneal exudates of mice injected with PBS or HA particles were re-stimulated *ex vivo* with cell culture medium or with heat-killed *Escherichia coli* (HK *E*. *coli*) for 24 hours, and the cytokines IL-1β, IL-10 and TNF-α were measured in supernatants to assess cytokine secretion. Figure 5C illustrates that following re-stimulation with HK *E*. *coli*, PECs from mice injected with N_5_ HA particles secreted significantly higher concentrations of the pro-inflammatory cytokine TNF-α when compared with PECs recovered from mice injected with PBS, which correlates well with the increased recruitment of immune cells in these groups. Furthermore, PECs from mice injected with N_5_, S_0.1_, R_20_ or S_20_ particles but not S_100_ particles showed a significantly diminished capacity to secrete IL-10, an immunoregulatory cytokine.

**Figure 6:**
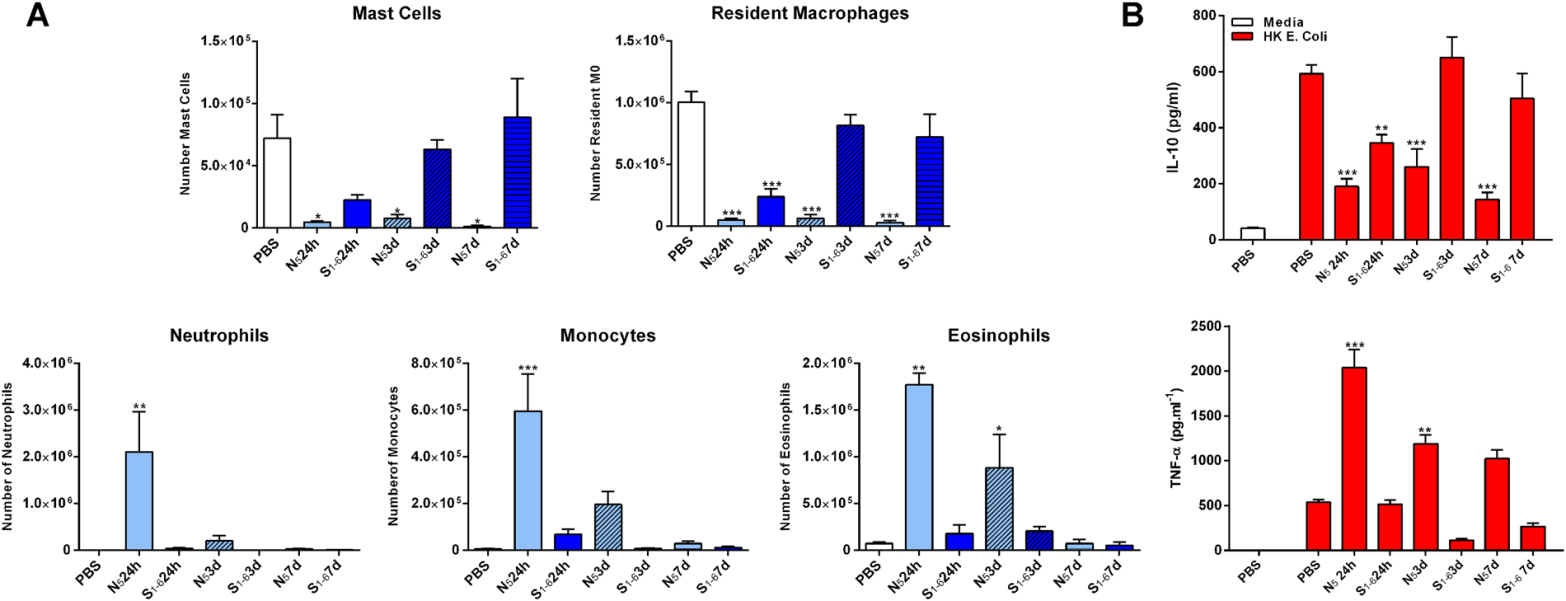
Injection of needle-shaped HA particles modulates cell recruitment *in vivo*. Female C57BL/6 mice (n = 3) aged 6–8 weeks were injected intraperitoneally with PBS vehicle, N_5_ (1 mg/mouse) or S_20_ 1–6 µm (1 mg/mouse) HA particles. Twenty-four hours, three days and seven days later peritoneal lavage cells were collected. (**A**) PECS were stained to analyse cell recruitment by flow cytometry. Cells were characterized using a combination of markers for peritoneal resident macrophages (CD11b^hi^ F4/80^hi^MHCII^−^), mast cells (SiglecF^−^ cKit^+^) monocytes (CD11b^+^ SiglecF^−^ F4/80^−^ Gr1int SSC^low^), neutrophils (CD11b^+^ SiglecF^−^ F4/80^−^ Gr1^hi^) and eosinophils (CD11b^+^ SiglecF^+^ Gr1^−^ SSC^hi^). Data are expressed as number of single/live cells. (*p < 0.05, **p < 0.01, ***p < 0.001). (**B**) PECs were restimulated with complete RPMI 1640 medium or heat-killed *Escherichia coli* (10 bacteria/cell) for 24 h. Supernatants were assessed for IL-10 and TNF-α secretion by ELISA. Results are mean cytokine concentrations (±SEM) for triplicate samples (vs PBS **p < 0.01, ***p < 0.001).

Finally, to further elucidate the effect of HA shape on the *in vivo* inflammatory response over time, we carried out an additional *in vivo* study with N_5_ and sieved S_20_ particles in the size range of 1–6 μm (S_1-6_). This allowed us to compare the inflammatory response to injection of spherical and needle-shaped particles of similar size over a 7 day period. As can be seen in Fig. 6A, significant differences were observed in inflammatory cell recruitment upon injection of N_5_ and S_1-6_ particles. One day after injection, needle-shaped particles led to resident macrophage depletion and a significant decrease in mast cells with a simultaneous recruitment of neutrophils, monocytes and eosinophils, which was not observed to the same extent with the spherical particles. Moreover, in mice injected with the spherical particles, mast cells and resident macrophages quickly repopulated the peritoneal cavity after 3 days, returning to homeostatic levels, suggesting that these particles promote less severe inflammatory responses compared to their needle-shaped counterparts. Interestingly, even after 7 days, the number of resident macrophages and mast cells was still significantly decreased in mice injected with N_5_ particles compared to PBS injected mice. In line with our previous results, re-stimulated PECs from mice injected with needle-shaped particles had an impaired capacity to secrete IL-10 across all time-points and produced significantly higher concentrations of TNF-α that declined over-time (Fig. 6B). This again suggests that the needle-shaped particles sustain an inflammatory environment even 7 days after injection. Overall, our results indicate that HA particle size and shape can be tailored to control the inflammatory response in order to promote favorable outcomes after implantation.

## Discussion

Host responses to implanted biomaterials are complex and it is vital to understand how biomaterials interact with the immune system in order to achieve successful tissue regeneration. Emerging evidence has highlighted the importance of material physicochemical characteristics in dictating the magnitude and type of immune responses induced, thereby presenting the opportunity to design biomaterials that can promote favorable immune cell recruitment and tissue remodeling^17, 28, 31^.

In this study, we investigated the relationship between the size, shape and surface topography of hydroxyapatite particles and their role in modulating the innate immune response. Five groups with similar composition but distinct characteristics (S_0.1_, N_5_, S_20_, R_20_, S_100_) were compared. Both *in vitro* and *in vivo* results demonstrate that HA particles differentially modulate cytokine secretion and cell recruitment, and that size and particularly morphology, but not surface topography, are key features in mediating this response. SEM and confocal images show that BMDCs were able to internalize considerable amounts of smaller S_0.1_ and N_5_ particles, and to a lower extent the larger S_20_ and R_20_ particles (Fig. 1B,C), and this correlates with the extent of IL-1β secretion. Moreover, we show that the secretion of IL-1β *in vitro* in response to particle formulation is dependent on the activation of the NLRP3 inflammasome. Finally, we demonstrate *in vivo* in an intraperitoneal mouse model that smaller needle-shaped particles increase the recruitment of immune cells and drive a sustained pro-inflammatory response. Taken together, the study highlights the importance of hydroxyapatite particle size and morphology, providing valuable indicators of the biomaterial parameters regulating immune outcomes for use in biomedical applications.

When studying the immunomodulatory effects of a biomaterial, it is crucial to report the level of endotoxin contamination or to show that the materials are not contaminated with endotoxins, to ensure that the inflammatory response is due to the material properties and not due to the presence of contaminants. Tynan *et al*.^26^ have shown that LPS concentrations as low as 10 pg · ml^−1^ are sufficient to induce BMDC secretion of the pro-inflammatory cytokine IL-6. In our study, particles alone failed to induce IL-1β secretion in BMDCs and BMDMs (Figs 2A and 3A) or IL-6 secretion in DCs (Fig. S1A), excluding significant endotoxin contamination in the preparations. Our observations contradict earlier reports that suggest a partial involvement of TLR-4 in the inflammatory response induced by HA particles^32^ and their ability to induce secretion of the pro-inflammatory cytokine TNF-α by macrophages^32, 33^. These dissimilarities may be related to differences in the cell types used but endotoxin contamination may be a key factor.

Our results demonstrate that, in addition to size, particle morphology greatly influences both internalization and the subsequent inflammatory response, with needle-shaped N_5_ particles eliciting stronger pro-inflammatory responses than their spherical counterparts, especially in macrophages. Indeed, N_5_ particles possess a higher aspect ratio (AR) when compared to the spherical S_0.1_ particles, which correlates well with higher secretion of IL-1β from cells in contact with these particles (Figs 2A and 3A). These observations are in line with other studies showing that smaller particles and particles with a higher AR are more readily phagocytozed^34, 35^. Interestingly, Vain *et al*.^36^ also observed a correlation between shape and inflammasome activation. Only budding particles (7–8 μm), but not their spherical counterparts, were able to induce significant IL-1β secretion in murine macrophages. Additionally, needle-shaped N_5_ particles also led to a significant decrease in viability at higher concentrations, in both DCs and macrophages, which could also be attributed to the shape of the particles, as several studies have reported that needle-shaped particles puncture the cell membrane, leading to increased toxicity. This has been established for needle-shaped particles of various compositions such as hydroxyapatite^37, 38^, polystyrene^39^ and silver^40^. The cytotoxic effects observed at high N_5_ concentrations could also be due to the release of reactive oxygen species during phagocytosis, a mechanism used by phagocytic cells to kill microorganisms^41^. Indeed, we show that inhibition of ROS production leads to a dose-dependent decrease in IL-1β secretion only in the case of needle shaped N_5_ particles (Fig. 4B).

Particle size is an intrinsic characteristic that influences the interaction of cells with biomaterials^18, 42^. Nanomaterials are generally described as being more immunostimulatory than micron-sized particles of the same material, when tested at the same weight. However, the same might not be true when relating to surface area, as cells directly interact with the surface area presented to them^43^. Using surface area relationships to normalize the dose of particles, we were able to demonstrate that the differences observed in terms of inflammatory profile generated by HA particles were due to differences in size rather than surface area presented. To further confirm the size-dependency, we decreased the polydispersity of the formulations and showed that IL-1β secretion by BMDCs is highest for smaller size particles (1–6 µm), moderate for medium size particles (15–20 µm) and negligible for particles larger than 30 μm (Fig. 2D). When compared side-by-side, needle-shaped particles in the range of 1–6 µm secreted significantly higher concentrations of the pro-inflammatory cytokine IL-1β then their spherical counterparts (Fig. S3). This clearly demonstrates a strong correlation between HA particle size and shape and inflammatory cytokine production. Jin and colleagues^29^ found that only irregular shaped particles are involved in the pathologic effect of phosphocalcic crystals, while we show that using a more discrete size range, spherical particles were also able to stimulate potent secretion of the proinflammatory cytokine IL-1β by dendritic cells. Interestingly, we did not observe any differences in the extent of IL-1β secretion between the two surface topographies tested (smooth S_20_ and rough R_20_), suggesting that size plays a greater role in determining pro-inflammatory responses compared to surface topography.

Moreover, our results prove that IL-1β secretion from BMDC in response to particles is entirely NLRP3 inflammasome dependent, as BMDCs derived from NLRP3^−/−^ mice did not secrete detectable concentrations of IL-1β when treated with HA particles (Fig. 4A). This is in agreement with previous reports that have shown that particulate material activates the NLRP3 inflammasome^31^. Our data show that distinct mechanisms are involved in inflammasome activation by HA particles (Fig. 4B). Even though particle uptake and lysosomal rupture are implicated in particulate-induced inflammasome activation, this is not always required. In fact, while lysosomal destabilization seems to be required to a certain extent for optimal IL-1β secretion in response to small N_5_ particles, the same was not observed for larger S_20_ particles. Chu and colleagues^44^ propose that sharp particles could rupture the endosome membrane and escape to the cytoplasm before lysosome formation, which could explain the lack of a dose-dependent reduction in IL-1β secretion following inhibition of lysosomal acidification. More strikingly, the ROS inhibitor DPI had a significant suppressive effect on IL-1β secretion triggered by needle-shaped particles but not by spherical particles. Studies suggest that particles with a higher AR result in “frustrated phagocytosis” that triggers ROS production, oxidative stress and subsequently inflammasome assembly^45, 46^. These data also correlate with the greater cytotoxicity observed for N_5_ particles. Moreover, we show that cathepsin B is required for NLRP3 inflammasome activation by HA particles, since its inhibition nearly abolished IL-1β production in a concentration-dependent manner for N_5_ and S_20_ particles. Overall, our data suggest that NLRP3 inflammasome activation by both N5 and S20 particles is dependent on cathepsin B release; however, ROS production only contributed to IL-1β secretion in the case of DCs stimulated with needle-shaped particles.

Whereas previous reports have addressed how HA physicochemical characteristics influence cell behavior *in vitro*, how these parameters impact on inflammatory responses following injection/implantation has received less attention. Under homeostatic conditions, the peritoneal cavity hosts a mixture of immune cell subsets^47, 48^. When a foreign material enters the peritoneum, it is sensed by innate cells, including resident macrophages capable of secreting cytokines and chemokines that drive the recruitment of neutrophils and monocytes^49^. In this study, in agreement with our *in vitro* data, we observed that injection of needle-shaped HA particles triggered the greatest inflammatory response, with respect to immune cell recruitment and the modulation of cytokine secretion (Fig. 5). Conversely, the largest S_100_ particles did not enhance IL-1β secretion *in vitro* or lead to significant immune cell infiltration *in vivo*, although a depletion of resident macrophages was observed. This suggests that although these particles are sensed by local cells, they do not elicit a significant inflammatory response due to their large size.

Velard *et al*.^50^ have shown *in vitro* that neutrophils treated with HA particles (size ranging from 100 nm to 10 μm) secrete cytokines (IL-1α and IL-8) and chemokines (MIP-1α and MIP-1β) that contribute to leukocyte chemotaxis. We envisage that HA particles modulate chemoattractant production *in vivo* in a manner dependent on particle size and shape, leading to differential cell recruitment. It has also been shown that TNF-α is involved in sustaining and enhancing neutrophil recruitment into sites of inflammation^51^. Indeed, upon restimulation *ex vivo*, we observed significant TNF-α production only in PECs of mice injected with N_5_ particles. Recent research by Sadtler *et al*.^52^ reported that Th2 cells are essential to generate a regenerative microenvironment surrounding a biomaterial scaffold. IL-10 functions as a regulatory cytokine, controlling the ability of APCs to regulate T cell responses^53^. Interestingly, we observed that IL-10 secretion after restimulation was nearly proportional to particle size.

Several reports have suggested that a strong pro-inflammatory response in the early stages of healing is essential for effective remodeling^54^, hence analyzing later time-points would help understand the role of particle size and morphology in long term remodeling outcomes. Our study shows that contrary to S_1-6_ particles, where the peritoneal cell composition returned to that seen in control mice 3 days after injection, injection of N_5_ particles mediated a long-lasting depletion of mast cells and macrophages while at the same time driving a transient recruitment of inflammatory immune cells (neutrophils, monocytes, and eosinophils), with similar kinetics to the vaccine adjuvant alum^55^. It is known that the number of resident macrophages declines during acute inflammation, a process referred to as “macrophage disappearance reaction”^56^. The basis for this macrophage depletion is still debatable, but has been attributed to cell death, increased adherence to the peritoneal cavity and migration to the draining lymph nodes^57^. Moreover, due to differences in the relative proportions of immune cells present in the peritoneal cavity, PECs recovered from mice injected with needle-shape particles secreted significantly lower amounts of IL-10, across all time-points, while the concentrations of the pro-inflammatory cytokine TNF-α remained significantly higher for at least 3 days.

Overall, our data suggest that incorporation of smooth and larger sized particles into biomaterials scaffolds might prove beneficial in promoting a tissue regenerative microenvironment upon implantation. This work highlights the importance of hydroxyapatite particle morphology and size in modulating the initial immune response after injection. Ongoing studies are aimed at elucidating the role of incorporation of these particles in biological scaffolds. Taken together, this study provides clear evidence for the pivotal role of intrinsic HA particle properties including shape and size in directing innate immune responses and presents the opportunity to fine-tune the host response by controlling the physical properties of materials.

## Conclusion

This study sheds light on the relationship between the physical properties of HA particles and the ensuing immune response. We provide evidence that HA particle size and morphology influence the production of inflammatory cytokines both *in vitro* and *in vivo*. Smaller needle-shaped HA particles generated a prolonged inflammatory response compared to spherical shaped nanoparticles and larger sized spherical HA particles, which suggest that these might be detrimental in promoting successful tissue remodeling. These findings indicate that by selecting distinct HA particles properties, tissue engineered constructs can be tailored to modulate the immune cell response to help provide regenerative signals to surrounding tissues.

### Experimental section

#### Reagents

Lipopolysaccharide from E. coli, Serotype R515, Toll-like receptor grade was purchased from Enzo Life Sciences. Aluminium hydroxide gel (Brenntag Biosector) is referred to in the manuscript as ‘alum’. Poly(deoxyadenylic-deoxythymidylic) acid, the cathepsin B inhibitor Ca-047-Me and DPI were obtained from Sigma. Endotoxin free water was purchased from Baxter Healthcare.

### Characterization of micron-sized hydroxyapatite particles

Hydroxyapatite particles of three different sizes and two different morphologies were purchased from Plasma Biotal Limited (Table 1). Mean micro-particle size of N_5_, S_20_, R_20_, and S_100_ was characterized using dynamic light scattering (DLS) (Malvern MasterSizer Sirocco 2000). ζ-potential was measured by electrophoretic light scattering (ELS) (Malvern ZetaSizer 3000).

### Synthesis and characterization of nano-sized hydroxyapatite particles

Nano-sized spherical, smooth hydroxyapatite particles (S_0.1_) were prepared following a previously described protocol^58^. Briefly, an aqueous solution of calcium chloride was slowly added to a solution of sodium phosphate and stirred for 2 min to promote precipitation of S_0.1_ particles. The solution was centrifuged and reconstituted in endotoxin-free water, sonicated for 10 min and passed through a 200 nm filter to remove any aggregated particles. Hydrodynamic size and ζ-potential of S_0.1_ particles were measured using DLS and ELS, respectively.

### Surface area measurement of microparticles

Specific surface area of the samples was determined by the nitrogen adsorption BET method using a Micromeritics Gemini VI (Micromeritics). Samples were prepared by purging under nitrogen overnight at 30 °C. Each sample was analyzed at least in duplicate.

### Scanning electron microscopy imaging to visualize hydroxyapatite particles

Samples were deposited on transparent adhesive strips or on glass coverslips placed on top of disposable stubs, and then sputter coated with gold/palladium using a Polaron Sputter Coater. Biological samples were prepared using alcohol dehydration and critical point drying (Quorum Technologies) before sputter coating. S_0.1_ particles were observed using cryo-SEM, where samples were plunged into nitrogen slush, freeze fractured, etched and sputter coated. Imaging was carried out using a Zeiss Ultra Plus FE (field emission) SEM operated at 5 KV in secondary electron mode.

### Mice

Female C57BL/6 mice were obtained from Harlan Olac (Bicester) and used at 8–16 weeks of age. NLRP3^−/−^ mice were bred in Trinity Biomedical Sciences Institute (TBSI) Comparative Medicines Unit. Animals were maintained according to the regulations of the European Union and the Irish Department of Health (Reference Number 091210). All animal studies were approved by the Trinity College Dublin Animal Research Ethics Committee.

### Murine cell cultures

Murine bone marrow-derived dendritic cells were generated as described previously^59^. Briefly, bone marrow cells were isolated from tibiae and femora of mice. Cells were grown in RPMI 1640 medium (Biosera) supplemented with 8% ultra-low endotoxin heat-inactivated foetal bovine serum (FBS) (Biosera), 2 mM L-glutamine (Gibco), 50 U · mL^−1^ penicillin (Gibco), 50 μg · mL^−1^ streptomycin (Gibco) and 20 ng · mL^−1^ of granulocyte–macrophage colony-stimulating factor (GM-CSF) derived from the J588 myeloma cell line. On day 10, the loosely adherent cells were harvested and plated at a density of 0.625 × 10^6^ cells · mL^−1^ and primed after 2 hours with LPS prior to stimulation with either alum or HA particles as indicated in figure legends. Supernatants were collected and analyzed for cytokine secretion and cells were harvested for flow cytometry analysis.

To obtain BMDMs, cells isolated from tibiae and femora of mice were grown in RPMI 1640 medium supplemented with 8% ultra-low endotoxin heat-inactivated foetal bovine serum (FBS), 2 mM L-glutamin, 50 U · mL^−1^ penicillin, 50 μg · mL^−1^ streptomycin and 20 % of L929 cell line conditioned medium containing macrophage colony-stimulating factor (M-CSF). On day 5, adherent cells were harvested and plated at a density of 1.0 × 10^6^ cells · mL^−1^ and left overnight before priming with LPS prior to stimulation with HA particles as indicated in figure legends. Supernatants were collected and analyzed for cytokine secretion.

### Cell viability in bone-marrow derived dendritic cell and bone-marrow derived macrophages

For the analysis of cellular viability, cells were incubated with fixable Aqua dead cell stain (Invitrogen) and subsequently stained with anti-CD11c (BD Pharmingen) to identify dendritic cells. Samples were acquired on BD FACSCanto II and the data analyzed using FlowJo software (Tree Star Inc.). Cellular viability in BMDMs was assessed using the lactate dehydrogenase (LDH) assay (Pierce), according to manufacturer’s instructions.

### Confocal microscopy analysis

HA particles were labeled with fluorescein isothiocyanate (FITC) at a ratio of 1:4 (1 mg of FITC per 4 mg of particles) for 18 h at 4 °C and washed twice with excess PBS to remove any unbound FITC. BMDCs from wild-type C57BL/6 mice (0.625 × 10^6^ cells · ml^−1^) were cultured on glass coverslips for 24 h with FITC-labeled HA particles (100 μg · ml^−1^). Cells were fixed with 4% paraformaldehyde for 10 min and stained for actin with 250 ng · ml^−1^ of phalloidin–tetramethylrhodamine B isothiocyanate (TRITC) and 4′,6-diamidino-2-phenylindole (DAPI) for nuclear staining for 30 min. Cells were mounted onto glass slides with mounting medium (Dako) and analysed on a confocal laser scanning platform Leica TCS SP8 with LSM 5 software.

### Innate immune responses to injection of hydroxyapatite particles

Mice were injected intraperitoneally (i.p.) with 200 μl of either PBS (Biosera) vehicle or HA particles (1 mg/mouse). After defined time periods, peritoneal exudate cells were collected by washing the peritoneal cavity of mice with 5 mL of PBS. PECs were pelleted by centrifugation (400 *g*, 5 min, 4 °C) and resuspended in complete RPMI.

In order to determine the nature of infiltrating cells, peritoneal lavage cells were stained as previously described^60^. Briefly, PECs were stained with Aqua dead cell stain, and subsequently stained with the following fluorescently labeled antibodies (eBiosciences, Biolegend or BD Pharmingen): CD11b, Gr1, F4/80, Siglec F, cKit, MHCcII. A Fortessa (BD Biosciences) flow cytometer was used, and data were analyzed using FlowJo software. Results were expressed as number of single/live cells.

For *ex vivo* analysis of cytokine production, PECs were plated at 1.0 × 10^6^ cell · ml^−1^ and stimulated with complete RPMI 1640 medium or heat-killed *Escherichia coli* (HK *E*. *coli*) (10 bacteria/cell). Supernatants were collected after 24 h and stored at −20 °C until further analysis.

### Cytokine ELISA

The concentration of the cytokines IL-6 and IL-10 was measured by ELISA using antibodies obtained from Biolegend. IL-1β, and TNF-α concentrations were also determined by ELISA according to the manufacturer’s instructions (R&D Systems).

### Statistics

Cytokine concentrations measured by ELISA assays were subjected to ANOVA analyses. Where significant differences were found, the Dunnett’s multiple comparisons post hoc test was performed. Statistical calculations were performed using the GraphPad Prism 6 software (GraphPad Software Inc.). In all cases, p-values ≤ 0.05 were considered statistically significant (*p < 0.05, **p < 0.01 and ***p < 0.001).

## Electronic supplementary material

Supplementary info
