# Supplementary material for: The shape and size of hydroxyapatite particles dictate inflammatory responses following implantation
Source: Sci Rep. 2017 Jun 7;7:2922. doi: 10.1038/s41598-017-03086-0 (PMC5462791; doi:10.1038/s41598-017-03086-0)
Supplement: Supplementary file 1 — Supplementary info [file 41598_2017_3086_MOESM1_ESM.pdf]

The shape and size of hydroxyapatite particles dictate inflammatory responses following implantation

Supplementary data

*Filipa Lebre‡1,4,5; Rukmani Sridharan‡2,3,5; Michael J. Sawkins2,3,5; Daniel J. Kelly2,3,5; Fergal J. O'Brien2,3,5; Ed C. Lavelle*1,4,5*

1 Adjuvant Research Group, School of Biochemistry and Immunology, Trinity Biomedical Sciences Institute, Trinity College Dublin, Dublin 2, D02 PN40, Ireland
2 Trinity Centre for Bioengineering, Trinity Biomedical Sciences Institute, Trinity College Dublin, Dublin 2, D02 PN40, Ireland
3 Tissue Engineering Research Group, Department of Anatomy, Royal College of Surgeons in Ireland, Dublin 2, Ireland
4 Centre for Research on Adaptive Nanostructures and Nanodevices (CRANN) Trinity College Dublin, Dublin 2, D02 PN40, Ireland
5 Advanced Materials Bio-Engineering Research Centre (AMBER), Trinity College Dublin, Dublin 2, D02 PN40, Ireland

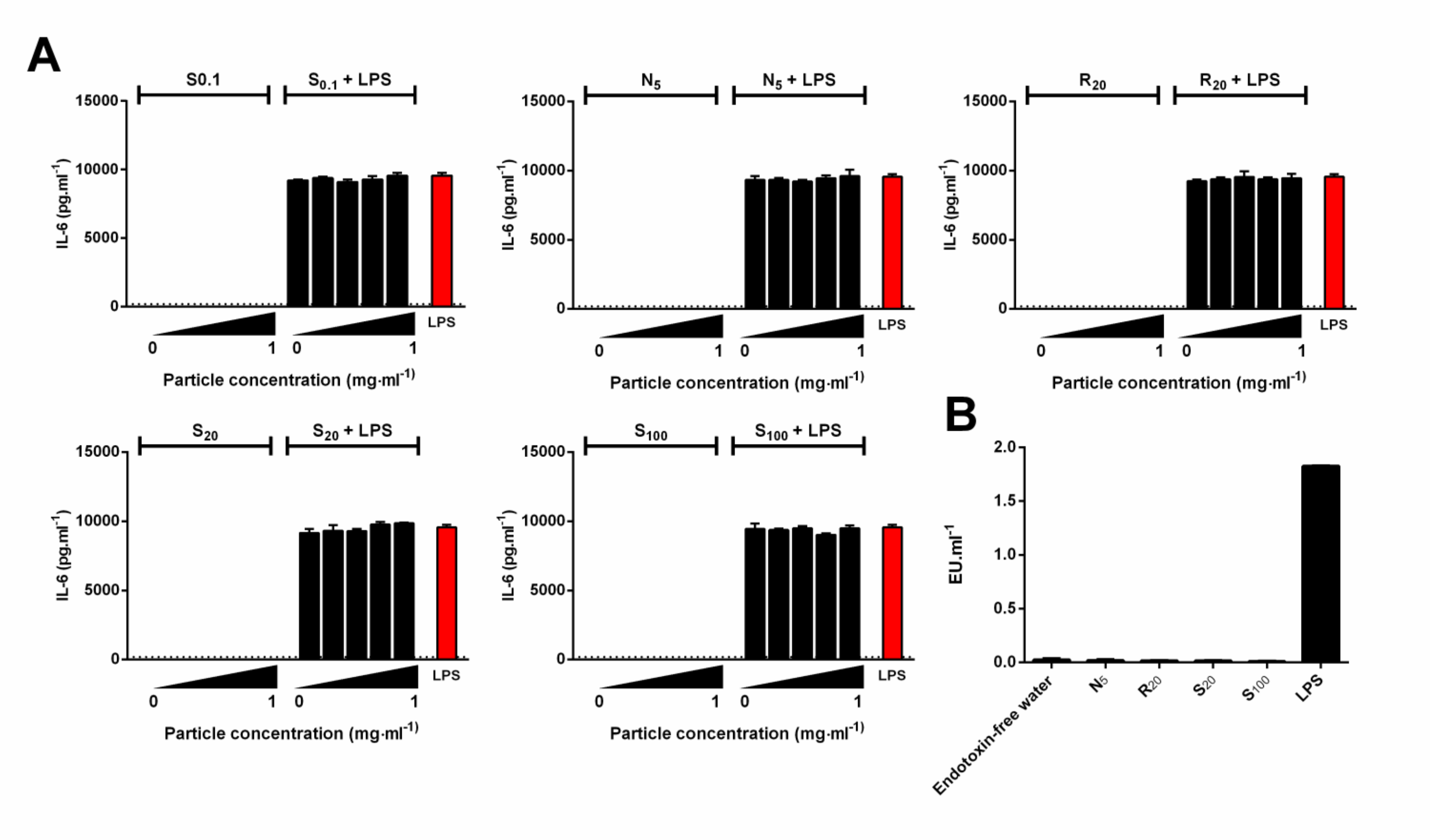

Supplementary Figure 1. Hydroxyapatite particle did not contain detectable endotoxin. (a) BMDCs (0.625 x 10^6 cells·ml^{-1}) from C57BL/6 mice were stimulated with concentrations ranging from 0.2 µg·ml^{-1} to 1 µg·ml^{-1} alone or after priming with LPS (1 ng·ml^{-1}) for 3 h. Supernatants were collected 24 h later and tested for IL-6 by ELISA. Results are mean cytokine concentrations (± SD) for triplicate samples. Data is representative of three independent experiments. (b) HA particles were tested for gram-negative bacterial endotoxins using LAL Chromogenic Endotoxin Quantitation Kit as per manufacturer’s instructions.

A

Sample Name		Particle Size (µm)		
		d(0.1)	*d(0.5)*	*d(0.9)*
S_{20}	>30 µm	23.94	36.91	57.81
	15-20 µm	11.03	15.76	22.33
	1-6 µm	8.99	12.88	18.29
R_{20}	>30 µm	27.13	37.98	52.84
	15-20 µm	10.56	15.17	21.55
	1-6 µm	7.08	10.41	15.13

B

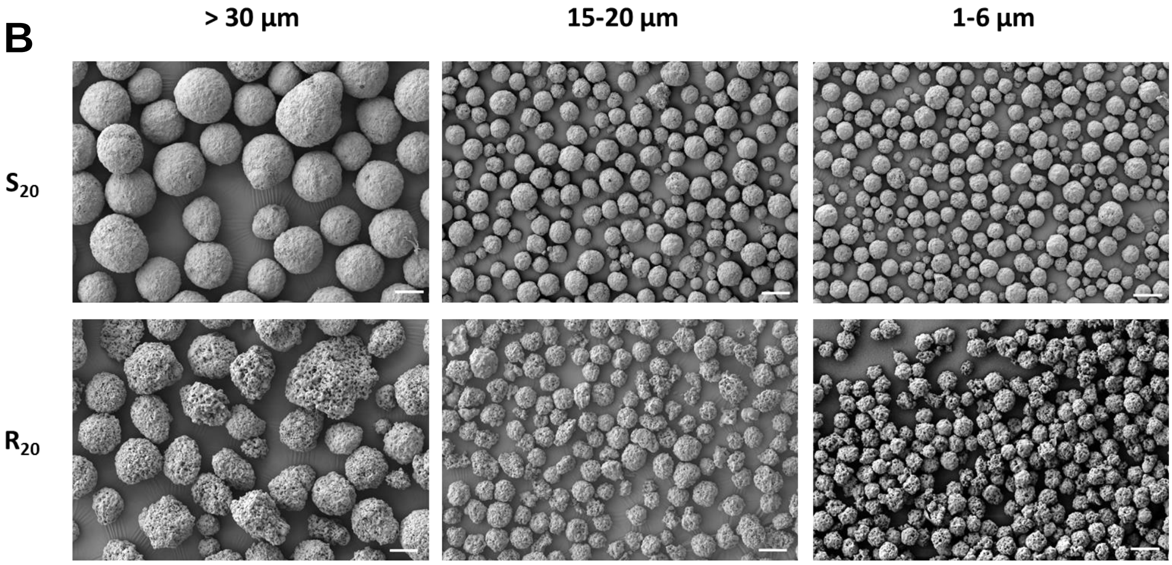

Supplementary Figure 2. Hydroxyapatite particles characterization after sieving. Size of HA particles was measured by dynamic light scattering (a); Scanning electron microscopy photomicrographs of S_{20} and R_{20} particles after sieving (PluriSelect) (b).

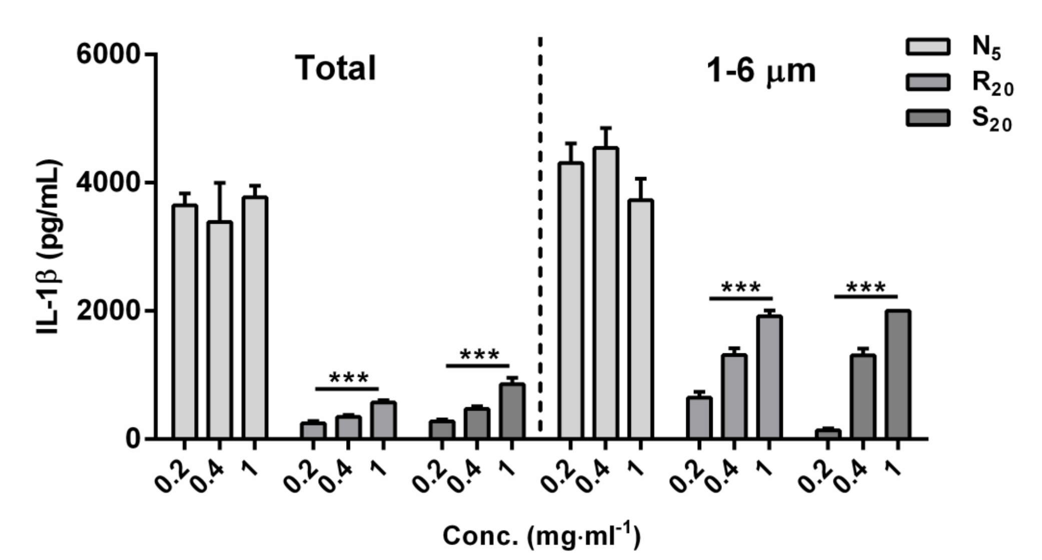

Supplementary Figure 3. Hydroxyapatite particle size and shape dictates cytokine production by murine BMDCs. BMDCs (0.625 x 10^6 cells·ml^{-1}) from C57BL/6 mice were stimulated with unsieved (total) and sieved N_5 particles (1-6 μm; 15-20 μm; > 30 μm), with concentrations ranging from 0.2 $mg.ml^{-1}$ to 1 $mg.ml^{-1}$ alone or after priming with LPS (1 ng·ml^{-1}) for 3 h. Supernatants were collected 24 hours later and tested for IL-1β by ELISA. Results are mean cytokine concentrations (± SD) for triplicate samples (vs N_5 ∗ $p < 0.05$, ∗∗ $p < 0.01$, ∗∗∗ $p < 0.001$). Data is representative of three independent experiments.

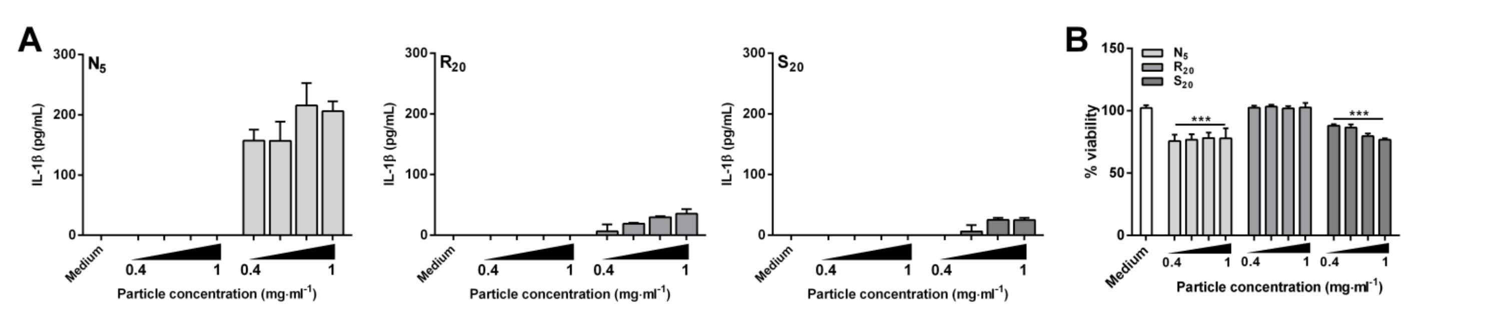

Supplementary Figure 4. Hydroxyapatite particle shape dictates cytokine production by murine BMDMs. BMDMs (1.0 x 10^6 cells·ml^{-1}) from C57BL/6 mice were stimulated with sieved particle (1-6 μm fraction) at concentrations ranging from 0.4 $mg.ml^{-1}$ to 1 $mg.ml^{-1}$ alone or after priming with LPS (1 ng·ml^{-1}) for 3 h. Supernatants were collected 24 hours later and tested for (**A**) IL-1β secretion by ELISA or (**B**) LDH release to assess cell viability. (A) Results are mean cytokine concentrations (± SD) for triplicate samples. Data are representative of three independent experiments. (B) Error bars show means ± SD for triplicate samples (vs medium ∗∗∗ $p < 0.001$). Results are representative of two independent experiments.

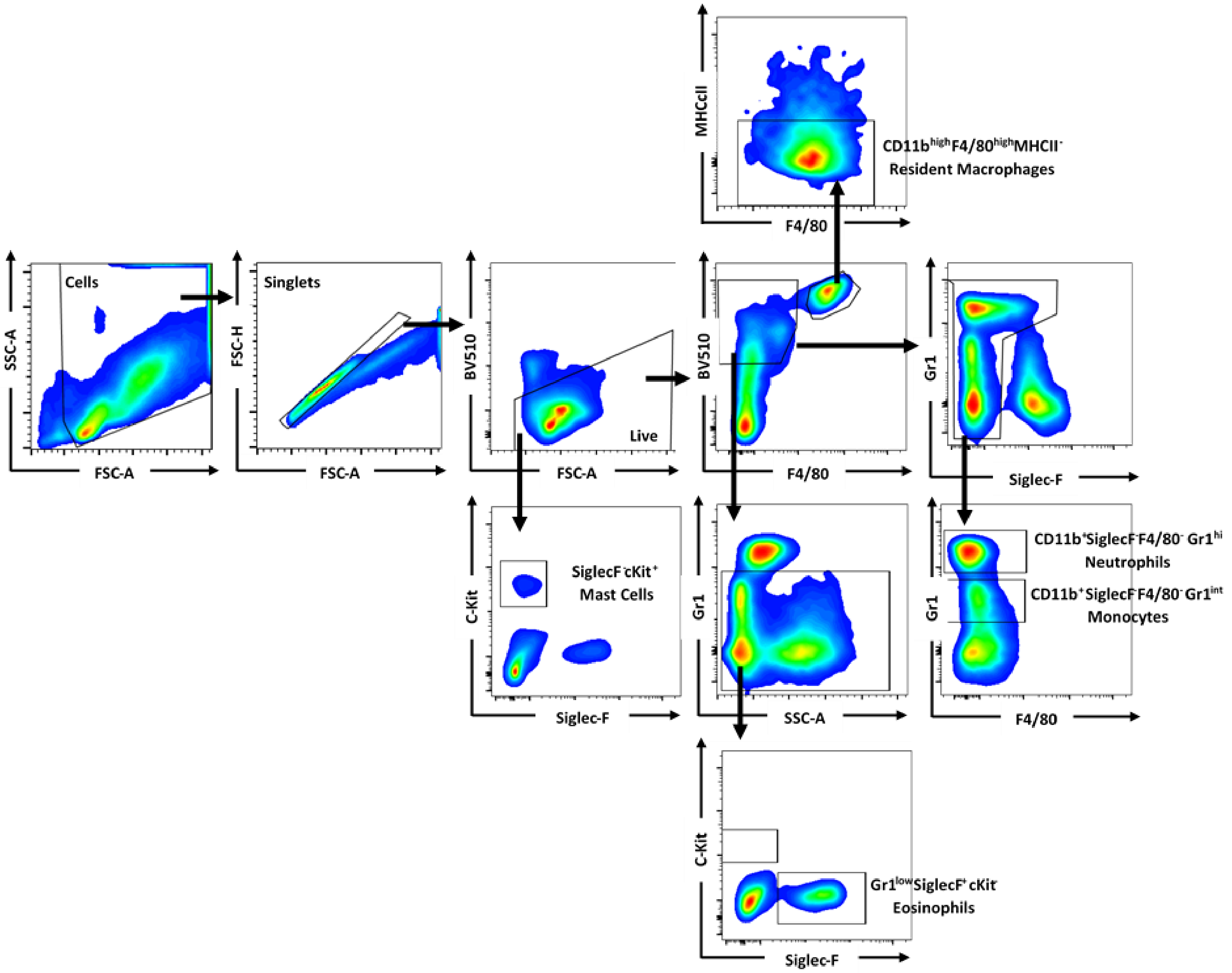

Supplementary Figure 5. Gating strategy for innate cell recruitment.
